# Impact of cavity shaving on residual tumor rates in patients with primary invasive carcinoma and carcinoma in situ in breast conserving surgery

**DOI:** 10.1007/s00404-022-06803-x

**Published:** 2022-10-25

**Authors:** Miriam Fernández-Pacheco, Michael Gerken, Olaf Ortmann, Atanas Ignatov, Monika Klinkhammer-Schalke, Maria Eleni Hatzipanagiotou, Elisabeth C. Inwald

**Affiliations:** 1 Department of Gynecology and Obstetrics, University Medical Center Regensburg, Regensburg, Germany; 2 Tumor Center - Institute for Quality Management and Health Services Research, University of Regensburg, Regensburg, Germany; 3 Bavarian Cancer Registry, Regional Centre Regensburg, Bavarian Health and Food Safety Authority, Regensburg, Germany; 4 Department of Gynecology and Obstetrics, Otto-Von-Guericke University, Magdeburg, Germany

**Keywords:** Breast conserving surgery, Cavity shaving, Invasive breast cancer, Re-excision rates, Residual tumor

## Abstract

**Background:**

Several international studies reported relatively high re-excision rates due to residual tumor in breast conserving surgery (BCS). Cavity shaving (CS) is a surgical strategy to reduce re-excision rates. This study aimed to investigate the effect of circumferential cavity shaving during BCS to reduce residual tumor.

**Material and Methods:**

A total of 591 patients with early invasive carcinoma or carcinoma in situ of the breast (ICD-10, C50 or D05) who were diagnosed between 01/01/2017 and 31/12/2019 and underwent BCS in a certified breast cancer center of the University Regensburg were analyzed regarding surgical excision methods. Patients with CS during BCS and patients with targeted re-excision in a specific direction depending on the result of intraoperative mammography or sonography during BCS were compared. The risk of pathologic residual tumor (R1) was compared between both groups by means of a multivariable binary logistic regression model to determine if there is a benefit of a certain surgical method to avoid a second intervention for re-excision. We adjusted for age, tumor size, nodal status, histologic type, surgeon, breast side, and neoadjuvant chemotherapy.

**Results:**

80 (*n* = 13.54%) patients had CS and 511 (*n = *86.46%) had a targeted re-excision in a specific direction during BCS according to intraoperative mammography or sonography. After comparing both techniques in a multivariable regression model, there was no significant difference regarding risk of residual tumor (*p* = 0.738) in the total cohort. However, CS showed a tendency to be favorable regarding rates of residual tumor in patients with invasive breast cancer between 60 and 70 years (*p* = 0.072) and smaller T1-tumors (*p* = 0.057) compared to targeted intraoperative re-excision following mammographic or sonographic assessment.

**Conclusion:**

CS showed a tendency to reduce residual tumor compared to the standard technique of intraoperative re-excision in specific subgroups, although no statistical significance was reached. Further studies are needed to overcome potential limitations like surgeon-based bias and missing standardized definitions of CS to reduce residual tumor rates.

## What does this study add to the clinical work

**Table:** 

In order to reduce re-excision rates in BCS, one of the available tools was CS, a surgical technique which showed a tendency for reduction of residual tumor rates. This benefit could only be shown in certain subgroups analyzed in this study.	

## Introduction

Breast cancer is the most prevalent cancer disease among women with 69.900 new cases in Germany in 2018 [1]. Breast-conserving surgery (BCS) followed by radiotherapy is predominantly performed in patients with early-stage breast cancer. The aim of surgical therapy is the R0 resection of the tumor [2].

BCS followed by radiotherapy of the entire breast is equivalent to mastectomy in terms of local recurrence and survival [3, 4]. Prerequisites for BCS instead of mastectomy are a favorable relation between tumor and breast size and localized tumors. In case of incomplete excision even after re-excision, inflammatory carcinoma or contraindication for radiotherapy after BCS mastectomy is mandatory [5, 6]. Cosmesis and patient satisfaction are important factors that have to be considered when offering BCS to patients. However, a disadvantage of BCS is the risk of positive margins, which occurs approximately in 20–40% of the cases after BCS [7]. Many attempts have been made to obtain clear margins of benign tissue around the carcinoma or carcinoma in situ [8–10]. The classical procedure is the excision of the tumor with further selective resections if necessary. If the tumor is not palpable, it will be needle-marked preoperatively either via mammography or via sonography. Another possibility to localize the tumor is the sonographic intraoperative imaging [11]***.*** Other endeavors to reduce residual tumor rates are margin assessment, i.e. via MarginProbe® or frozen section and pathological assessment [12]***.*** Another attempt to reduce margin positivity is ultrasound-guided surgery which could be potentially beneficial regarding margin status [13]. Beside standard needle marking, there are other options for marking such as radiofrequency identification (RFID), radioactive or magnetic seeds among others [14].

There is no consistent definition of positive or negative margins. According to the German interdisciplinary S3 Guideline for the Early detection, Diagnosis, Treatment and Follow up Care of Breast Cancer, a margin distance superior or equal to 2 mm is considered as R0 in case of carcinoma in situ [15]. If the margin is closer than 2 mm, indication for re-excision is given to achieve R0 status in carcinoma in situ [16]. In contrast, the margin distance has to be just one cell layer in invasive carcinoma to reach R0 status [17], i.e., no ink on tumor”.

Several studies showed the necessity of a second surgery for margin clearance in 20–30% of BCS cases [18]. Data from the quality report of the certified breast cancer centers in Germany showed re-excision rates of 15,04% in Germany in 2018 [19]. Some retrospective studies claimed that taking additional tissue circumferentially around the cavity left by initial tumorectomy reduces the rate of residual margins and re-excision rates [20, 21]. The technique of CS consists in the resection of all borders of the tumor bed in a circumferential way after regular tumor excision. The aim is the reduction of histological tumor bed positivity and of re-excision rates. Economic detriment and surgical complications might be lowered by CS and the patients’ compliance and satisfaction might increase. However, other studies showed that the standard procedure of excising selective margins where the tumor seems to be close to the specimen’s margin according to intraoperative mammographic or sonographic assessment may be sufficient for reaching R0 status [22]. Thus, results from studies are inconsistent. We performed a retrospective study to analyze the effect of cavity shaving in comparison to the standard procedure of mammography or sonography of the tumor specimen followed by re-excision if necessary analyzing data of a large cohort of patients treated in a certified German breast cancer center.

## Methods

### Database

The present retrospective analysis included 1067 patients from 18 years of age onwards with either breast cancer of stage 0 to III or carcinoma in situ who were diagnosed and treated between January 2017 and December 2019 in the certified breast cancer center of the University Medical Center in Regensburg, Germany.

Data of these patients from the regional population-based clinical cancer registry (Tumor Centre Regensburg, Bavaria, Germany) were analyzed. A population of more than 2.2 million people including Upper Palatinate and Lower Bavaria is covered in this population-based regional cancer registry. Electronic sheets of documentation contain information about diagnosis, course of disease, therapies, and the complete follow-up of patients. These population‐based data originate from medical reports, pathology reports and follow‐up records. Diagnosis, therapy modalities, course of disease and several histologic parameters are documented as well as long‐term follow‐up including locoregional or distant recurrence and mortality.

### Inclusion and exclusion criteria

The base-line cohort included 1067 cases of patients who obtained surgical treatment in the certified breast cancer center of the University Regensburg between 01/01/2017 and 31/12/2019. Female patients with either primary invasive carcinoma or carcinoma in situ of the breast (ICD-10 C50/D 05) from stage I–III and BCS with or without re-excision were included. Patients with neoadjuvant chemotherapy were included in our study as long as they did not have pT0‐tumors after BCS.

**Fig. 1:**
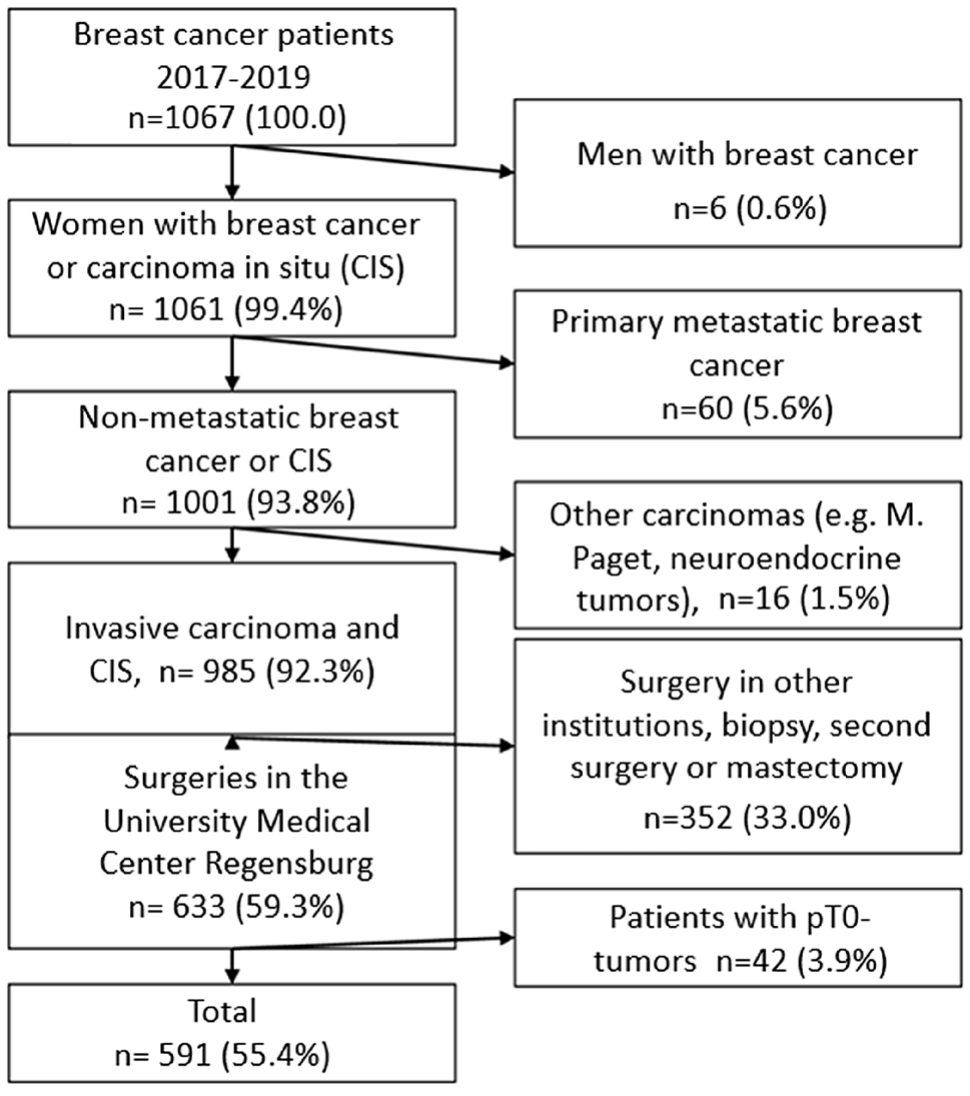
Flow chart

Exclusion criteria were male sex, primary metastatic breast cancer, unusual histological types such as Paget or neuroendocrine carcinoma, patients who had previous BCS in other institutions, and mastectomy or second surgery during our study period. Patients with T0-tumors after surgery were excluded. Finally, 55.4% (*n* = 591 patients) of our initial cohort fulfilled inclusion criteria and were eligible for analysis. The inclusion and exclusion criteria and the study design are presented in Fig. 1.

### Surgical technique and surgeons

In total data from *n* = 591 patients were analyzed regarding surgical technique, i.e. additional circumferential CS and selective resection of one or more margins after mammographic or sonographic intraoperative assessment of margin status. CS was defined as an additional circumferential excision of tissue around the initial lumpectomy cavity left by partial mastectomy, if possible as a single, circumferential piece to provide easy orientation for the examining pathologist. Totally, six surgeons performed breast surgery. CS was mainly conducted by one surgeon who systematically performed this technique in all BCS from January 2017 to December 2019. Five experienced surgeons, who were also trained in CS conducted most of their surgeries according to standard procedure. In non-palpable tumors, imaging-guided marking was performed preoperatively by sonography or mammography. After tumor excision, the removed tissue was examined intraoperatively either via sonography or mammography to confirm the completeness of the exstirpated tumor. If there was any imaging suspicion for the tumor being too close or at the specimen´s margin, the surgeon was informed to perform a selective re‐excision in that direction/s. The effect of surgical technique on histological outcome was analyzed by comparison of risk of R1 pathologic assessment in both groups. Furthermore, related to R1, the need of second surgery was compared between both groups of patients.

In sano resection (R0) was defined as a margin distance of at least one layer of tumor‐free cells in invasive carcinoma, i.e. “no ink on tumor”. Regarding DCIS, R0 resection was defined as a distance of at least 2 mm between DCIS and margins. Concerning the surgical aspect, preoperative needle marking of tumor by sonography or mammography, intraoperative sonography or mammography of tumor specimen, surgeon, need of re-excision and strategy of surgery were analyzed. These characteristics were compared between patients who obtained standard BCS and those who underwent BCS with CS.

### Statistical analysis

Continuous data were depicted as means, medians, and standard deviations (SD), categorical data were expressed as frequency counts and percentages. Comparison of means was performed by Student’s *t*‐test for normally distributed continuous variables (assessed by Kolmogorov–Smirnov test), otherwise by Mann‐Whitney *U* test. Pearson’s *χ*^2^ test was applied for testing the independence of categorical variables to compare the base-line characteristics of patients. A multivariable binary logistic regression analysis was performed to evaluate the influence of cavity shaving compared to standard BCS on the risk of residual tumor adjusting for confounding variables: breast side, menopausal status, histologic tumor type, associated DCIS, tumor size (T‐status), nodal status (N), grading, lymphatic invasion, vascular invasion, hormonal receptor status, HER2‐status, Ki67, neoadjuvant chemotherapy, preoperative marking of tumor, intraoperative sonography or mammography of extracted tissue, surgeon, need of re-excision, and strategy of surgery. The logistic regression odds ratio (OR) and corresponding 95% confidence interval (CI) were estimated and regarded as statically significant if the CI excluded 1.0. Listed *p* values from the log‐rank tests were two-sided and statistical results were regarded as significant at a *p*-value of < 0.05. Statistical analyses and calculations were conducted with the software package SPSS 25 (Chicago, IL, USA). Additional individual patient consent for this analysis was not needed. The manuscript was prepared in accordance with the statement criteria of STROBE (Strengthening the Reporting of Observational Studies in Epidemiology).

## Results

Among all 1067 initially registered patients from 01/01/2017 to 31/12/2019, only 0.6% (*n* = 6) were men and therefore excluded. 5.6% of (*n* = 60) patients were excluded due to primary metastatic breast cancer, 16 patients (1.5%) had unusual histological types such as Paget or neuroendocrine carcinoma and were also excluded. Furthermore, 352 patients (33.0%) who had previous BCS in other institutions or underwent mastectomy or second surgery during our study period were excluded. 37 patients (3.5%) had ypT0-tumors after neoadjuvant therapy were also excluded (Fig. 1). Finally, 591 patients (55.4%) of our initial cohort were eligible for analysis. Of the 591 patients analyzed, 511 patients (86.5%) underwent BCS with standard procedure and 80 patients (13.5%) underwent BCS with CS. Among 511 patients with standard BCS, 138 patients (27.0%) had positive margins in the final histologic assessment, whereas 21 patients (26.3%) with BCS with CS had residual tumor (*p* = 0.887). The clinical and pathological characteristics of the two surgery groups are shown in Table 1. Regarding age of patients, there was no significant difference between both groups (*p* = 0.138). The distribution of menopausal status was very similar between both groups, there were 8 premenopausal women (13.8%) in the CS group compared to 59 (15.0%) in the standard group (*p* = 0.138). More than two thirds of patients were postmenopausal in the CS and the standard group (69.0, *n* = 40 vs. 70.2%, *n* = 276). Regarding breast side, the distribution among both groups was also homogeneous, being 48.8% (*n* = 39) and 51.2% (*n* = 262) for the left side in the CS and standard group and 41% (*n* = 249) and 51.2% (*n* = 48) for the right side. Small tumors (T1) were more frequent compared to larger ones (T2‐T4). Comparing the CS and the standard group, 41.3% (*n* = 33) vs. 38.2% (*n* = 195) of patients had T1-tumors, whereas 30.0% (*n* = 24) vs. 32.3% (*n* = 165) of patients had T2-4 tumors. Still, statistical significance in distribution between groups was not reached in tumor size (*p* = 0.390). Almost all parameters had a homogeneous distribution between both groups. However, regarding nodal status, a significant difference between both groups (*p* = 0.035) was found. Patients in the group with CS had tumor-free lymph nodes in 53.8% of the cases (*n* = 43), in contrast to the group with standard procedure with 64.6% (*n* = 373) of patients. 18.8% of patients (*n* = 15) had nodal invasion in the CS group, versus 19.6% (*n* = 100) of the patients in the standard group. Histological type was the parameter which was closest to statistical significance with a p-value of *p* = 0,096. Invasive ductal carcinoma was the most frequent type in both groups with 75.0% (*n* = 60) in the CS group and 69.9% (*n* = 357) in the standard group. Though, invasive ductal carcinoma and invasive lobular carcinoma had a similar distribution (11.3%, *n* = 9 and 11.7% *n* = 60) in both groups. Other carcinomas such as Paget carcinoma were more frequent in the CS group (5.0%, *n* = 4 vs. 1.8%, *n* = 9). Ductal carcinoma in situ was almost twice as frequent in the group receiving standard BCS procedure (8.8%, *n* = 7 vs. 16.6%, *n* = 85) with a p-value of *p* = 0.096. Only 8,8% of the patients (*n* = 52) received neoadjuvant chemotherapy vs. 91.2% (*n* = 539) without neoadjuvant chemotherapy. The distribution among both groups was homogeneous (*p* = 0.595), as shown in Table 1. Regarding the five main surgeons who performed BCS, distribution concerning numbers of surgery and strategy of surgeons was heterogeneous. In patients receiving CS surgeon 1 dominated with in 61.3% (*n* = 49), surgeon 2, 3, 4, 5 and other surgeons performed 11.3% (*n* = 9), 0% (*n* = 0), 7.5% (*n* = 6), 6.3% (*n* = 5) and 13.8% (*n* = 11) CS. Standard BCS, on the other hand, was performed more often than CS among surgeons (2, 3, 4, 5 and others) than by surgeon 1, who performed 17.2% (*n* = 88) of standard BCS. Table 2 shows tumor and patients’ characteristics, as well as surgeon‐ and surgery‐ dependent parameters according to R1 or R0 status. Noteworthy, the percentage of premenopausal patients among R1‐status (12.8%, *n* = 42) was considerably higher in comparison with postmenopausal patients (65.6%, *n* = 80; *p* = 0.218). Regarding tumor characteristics, the proportion of ductal carcinoma in situ was much higher in tumors with affected specimen margins (21.4%, *n* = 34), among tumors with R0 status the proportion was 13.4% (*n* = 58). Size of tumor had also a different distribution among R0 and R1-resected specimens. Among R0 resected tumors T1-stage predominated, being 41.2% (*n* = 178), followed by T2 to 4-stage (29,9%, *n* = 129). On the other side, among tumors with final histopathological classification of R1, T2-4 stage was more prevalent with 37,7% (*n* = 60) in comparison to T1-stage with 31.4% (*n* = 50). Nodal positivity (25.8% vs. 17.1%, *p* = 0.062), lymphatic invasion (21.4% vs. 14.1%, *p* = 0.004) and vascular invasion (4.4% vs. 1.9%, *p* = 0.024) were more common in tumors with positive margins in comparison with R0-tumors. Tumors with margin positivity had a higher rate of hormone‐receptor negative tumors (10.1%, *n* = 16 vs. 6.3%, *n* = 27; *p* = 0.102), higher Ki67 rates (*p* = 0.002), and a lower rate of HER2‐negative tumors (69.2%, *n* = 110 vs. 80.3%, *n* = 347; *p* = 0.006). Table 3 shows the results from the multivariable, binary logistic regression for risk of R1 resection depending on surgical strategy CS vs standard procedure and patient characteristics. In the total cohort, the risk of R1 resection was very similar in patients receiving cavity shaving or standard procedure with an OR = 1.104 for CS vs. standard procedure (95% CI 0.620–1.965, *p* = 0.738). The risk of final R1-status was 50% lower in patients older than 70 years compared to patients younger than 50 years (OR 0.527; 95% CI, 0.277–1.003, *p* = 0.051). Furthermore, the risk of R1 resection was threefold higher for patients with DCIS compared to patients with invasive ductal carcinoma with an OR of 3.413 (95%, CI 0.723–16.109, *p* = 0.121). The risk for R1 resection was also higher for tumors with positive lymph node status (N1-3), OR = 1.580 (95% CI 0.894–2.791, *p* = 0.115). Regarding tumor biology, the risk for R1 resection was higher in hormone receptor negative tumors than in hormone receptor positive tumors, OR being 2.241 (95% CI 1.056–4.756, *p* = 0.036). HER2 negative tumors had a lower probability of having R1 status than the HER2 positive tumors, with an OR of 0.5000 (95% CI 0.251–0.996, *p* = 0.049). Table 4 summarizes results from univariable and multivariable binary logistic regression analyses concerning the risk of R1 status with cavity shaving versus standard procedure in the total cohort, in histologic subgroups, and in the subgroup of patients with invasive ductal carcinoma. Stratified analysis was restricted to invasive ductal carcinoma, since it had the largest cohort compared to other histologic subgroups. Due to the smaller size of cohorts, invasive lobular carcinoma or ductal carcinoma in situ were not analyzed. Regarding age, women aged 60 to 69 years had a benefit from cavity shaving, the risk of reaching R1 status being lower with an OR of 0.227 (95% CI 0.050–1.027), reaching almost statistical significance with a p-value of 0.054 in univariable logistic regression. A multivariable regression showed an OR of 0.168 (95% CI 0.024–1.171; *p* = 0.072). Smaller T1 tumors had a tendency for having R0-status with cavity shaving compared to standard procedure, OR being 0.247 (95% CI 0.056–1.090) in univariable logistic regression. Here the multivariable regression showed an OR of 0.214 (95% CI 0.044–1.05; *p* = 0.057). For other analyzed variables such as nodal status, grading, HER2neu status or associated DCIS no significant difference between both surgical methods regarding the risk of final R1-status was reached. In conclusion, women with invasive ductal carcinoma aged between 60 and 69 years and patients with small tumors seemed to have a slight benefit from cavity shaving regarding risk of residual tumor. Apart from that, there was no statistical difference between cavity shaving and standard procedure.

**Table 1:** Patients´ characteristics according to surgery strategy

		Strategy of surgery						
		Margin shaving		Standard surgical procedure		Total		
		*N*	%	*N*	%	*N*	%	p*
Age at diagnosis (years)	< 50	9	11.3%	93	18.2%	102	17.3%	
	50–59	19	23.8%	153	29.9%	172	29.1%	.138
	60–69	32	40.0%	151	29.5%	183	31.0%	
	≥ 70	20	25.0%	114	22.3%	134	22.7%	
Menopausal status	Premenopausal	8	13.8%	59	15.0%	67	14.9%	
	Perimenopausal	1	1.7%	5	1.3%	6	1.3%	.962
	Postmenopausal	40	69.0%	276	70.2%	316	70.1%	
	Ns	9	15.5%	53	13.5%	62	13.7%	
Histologic type	Inv. ductal carcinoma	60	75.0%	357	69.9%	417	70.6%	
	Inv. Lobular carcinoma	9	11.3%	60	11.7%	69	11.7%	.096
	Other carcinomas	4	5.0%	9	1.8%	13	2.2%	
	Carcinoma in situ	7	8.8%	85	16.6%	92	15.6%	
Side	Left	39	48.8%	262	51.3%	301	50.9%	.675
	Right	41	51.2%	249	48.7%	290	49.1%	
T pathologic (pT)	T1	33	41.3%	195	38.2%	228	38.6%	
	T2-4	24	30.0%	165	32.3%	189	32.0%	.390
	Tis	7	8.8%	74	14.5%	81	13.7%	
	Tx/ns	16	20.0%	77	15.1%	93	15.7%	
N pathologic (pN)	N0	43	53.8%	330	64.6%	373	63.1%	
	N1-3	15	18.8%	100	19.6%	115	19.5%	.035
	Nx/ns	22	27.5%	81	15.9%	103	17.4%	
Grading	G1	15	18.8%	111	21.7%	126	21.3%	
	G2	43	53.8%	238	46.6%	281	47.5%	.599
	G3	10	12.5%	61	11.9%	71	12.0%	
	GX/ns	12	15.0%	101	19.8%	113	19.1%	
Lymphvessel invasion	L0	51	63.7%	343	67.1%	394	66.7%	
	L1	14	17.5%	81	15.9%	95	16.1%	.838
	LX/ns	15	18.8%	87	17.0%	102	17.3%	
Vascular invasion	V0	64	80.0%	408	79.8%	472	79.9%	
	V1/2	1	1.3%	14	2.7%	15	2.5%	.714
	VX/ns	15	18.8%	89	17.4%	104	17.6%	
Hormonal receptor status	Positive	73	91.3%	461	90.2%	534	90.4%	
	Negative	6	7.5%	37	7.2%	43	7.3%	.777
	Ns	1	1.3%	13	2.5%	14	2.4%	
Her2/neu	Positive	6	7.5%	50	9.8%	56	9.5%	
	Negative	66	82.5%	391	76.5%	457	77.3%	.492
	Ns	8	10.0%	70	13.7%	78	13.2%	
Ki67	Low risk ≤ 15%	48	60.0%	328	64.2%	376	63.6%	
	High risk > 15%	23	28.7%	133	26.0%	156	26.4%	.766
	Ns	9	11.3%	50	9.8%	59	10.0%	
Associated ductal carcinoma in situ	Yes	12	15.0%	66	12.9%	78	13.2%	.609
	No	68	85.0%	445	87.1%	513	86.8%	
Associated lobular intraneoplasia	Yes	0	0.0%	2	0.4%	2	0.3%	.575
	No	80	100.0%	509	99.6%	589	99.7%	
Neoadjuvant chemotherapy	Yes	6	7.5%	46	9.0%	52	8.8%	.575
	No	74	92.5%	465	91.0%	539	91.2%	
Preoperative needle marking	Yes	67	83.8%	414	81.0%	481	81.4%	.561
	No	13	16.3%	97	19.0%	110	18.6%	
Intraoperative mammography	Yes	21	26.3%	164	32.2%	185	31.4%	.559
	No	59	73.8%	346	67.8%	405	68.6%	
Intraoperative sonography	Yes	56	70.0%	317	62.0%	373	63.1%	
	No	24	30.0%	194	38.0%	218	36.9%	.170
Surgeon	Surgeon 1	49	61.3%	88	17.2%	137	23.2%	
	Surgeon 2	9	11.3%	58	11.4%	67	11.3%	.000
	Surgeon 3	0	0.0%	52	10.2%	52	8.8%	
	Surgeon 4	6	7.5%	110	21.5%	116	19.6%	
	Surgeon 5	5	6.3%	84	16.4%	89	15.1%	
	Others	11	13.8%	119	23.3%	130	22.0%	
Residual tumor	R0	59	73.8%	373	73.0%	432	73.1%	
	R1	21	26.3%	138	27.0%	159	26.9%	.887
	Total	80	100.0%	511	100.0%	591	100.0%	

*p-value from Pearson‘s *χ*^2^ test

**Table 2:** Patient characteristics according to residual tumor

		Residual tumor						
		R0		R1		Total		
		*N*	%	*N*	%	*N*	%	p*
Age at diagnosis (years)	< 50	67	15.5%	35	22.0%	102	17.3%	
	50–59	122	28.2%	50	31.4%	172	29.1%	.111
	60–69	137	31.7%	46	28.9%	183	31.0%	
	≥ 70	106	24.5%	28	17.6%	134	22.7%	
Menopausal status	Premenopausal	42	12.8%	25	20.5%	67	14.9%	.218
	Perimenopausal	5	1.5%	1	0.8%	6	1.3%	
	Postmenopausal	236	71.7%	80	65.6%	316	70.1%	
	Ns	46	14.0%	16	13.1%	62	13.7%	
Histologic type	Inv. ductal carcinoma	312	72.2%	105	66.0%	417	70.6%	
	Inv. lobular carcinoma	52	12.0%	17	10.7%	69	11.7%	.131
	Other carcinomas	10	2.3%	3	1.9%	13	2.2%	
	Carcinoma in situ	58	13.4%	34	21.4%	92	15.6%	
Side	Left	229	53.0%	72	45.3%	301	50.9%	.096
	Right	203	47.0%	87	54.7%	290	49.1%	
T pathologic (pT)	T1	178	41.2%	50	31.4%	228	38.6%	
	T2-4	129	29.9%	60	37.7%	189	32.0%	.062
	Tis	54	12.5%	27	17.0%	81	13.7%	
	Tx/ns	71	16.4%	22	13.8%	93	15.7%	
N pathologic (pN)	N0	281	65.0%	92	57.9%	373	63.1%	
	N1-3	74	17.1%	41	25.8%	115	19.5%	.062
	Nx/ns	77	17.8%	26	16.4%	103	17.4%	
Grading	G1	93	21.5%	33	20.8%	126	21.3%	
	G2	211	48.8%	70	44.0%	281	47.5%	.613
	G3	50	11.6%	21	13.2%	71	12.0%	
	GX/ns	78	18.1%	35	22.0%	113	19.1%	
Lymphvessel invasion	L0	305	70.6%	89	56.0%	394	66.7%	.004
	L1	61	14.1%	34	21.4%	95	16.1%	
	LX/ns	66	15.3%	36	22.6%	102	17.3%	
Vascular invasion	V0	356	82.4%	116	73.0%	472	79.9%	
	V1/2	8	1.9%	7	4.4%	15	2.5%	.024
	VX/ns	68	15.7%	36	22.6%	104	17.6%	
Hormonal receptor status	Positive	397	91.9%	137	86.2%	534	90.4%	.102
	Negative	27	6.3%	16	10.1%	43	7.3%	
	Ns	8	1.9%	6	3.8%	14	2.4%	
Her2/neu status	Positive	39	9.0%	17	10.7%	56	9.5%	.006
	Negative	347	80.3%	110	69.2%	457	77.3%	
	Ns	46	10.6%	32	20.1%	78	13.2%	
Ki67	Low risk ≤ 15%	285	66.0%	91	57.2%	376	63.6%	
	High risk > 15%	115	26.6%	41	25.8%	156	26.4%	.002
	Ns	32	7.4%	27	17.0%	59	10.0%	
Associated ductal carcinoma in situ	Yes	49	11.3%	29	18.2%	78	13.2%	
	No	383	88.7%	130	81.8%	513	86.8%	.028
Neoadjuvant chemotherapy	Yes	41	9.5%	11	6.9%	52	8.8%	
	No	391	90.5%	148	93.1%	539	91.2%	.328
Preoperative needle marking	Yes	352	81.5%	129	81.1%	481	81.4%	
	No	80	18.5%	30	18.9%	110	18.6%	.923
Intraoperative mammography	Yes	123	28.5%	62	39.0%	185	31.4%	
	No	308	71.5%	97	61.0%	405	68.6%	.015
Intraoperative sonography	Yes	288	66.7%	85	53.5%	373	63.1%	
	No	144	33.3%	74	46.5%	218	36.9%	.003
Surgeon	Surgeon 1	106	24.5%	31	19.5%	137	23.2%	
	Surgeon 2	57	13.2%	10	6.3%	67	11.3%	
	Surgeon 3	31	7.2%	21	13.2%	52	8.8%	
	Surgeon 4	84	19.4%	32	20.1%	116	19.6%	.034
	Surgeon 5	64	14.8%	25	15.7%	89	15.1%	
	Others	90	20.8%	40	25.2%	130	22.0%	
Second surgery	No	325	98.8%	3	2.5%	328	72.7%	.000
	Yes	4	1.2%	119	97.5%	123	27.3%	
Strategy of surgery	Margin shaving	59	13.7%	21	13.2%	80	13.5%	
	Standard surgical procedure	373	86.3%	138	86.8%	511	86.5%	.887
	Total	432	100.0%	159	100.0%	591	100.0%	

*p-value from Pearson ‘s *χ*^2^ test

**Table 3:** Results from multivariable binary logistic regression for risk of R1 depending on surgical strategy and patients´ characteristics

	*p*	OR*	Lower 95%-CI	Upper 95%-CI
Margin Shaving no		1.000		
Margin Shaving yes	.738	1.104	.620	1.965
Age at diagnosis < 50 years		1.000		
Age at diagnosis, 50–59 years	.270	.719	.400	1.292
Age at diagnosis, 60–69 years	.423	.787	.439	1.413
Age at diagnosis, ≥ 70 years	.051	.527	.277	1.003
Invasive ductal carcinoma		1.000		
Invasive lobular carcinoma	.592	1.195	.624	2.290
Other carcinomas	.825	1.167	.295	4.612
Carcinoma in situ	.121	3.413	.723	16.109
Left side		1.000		
Right side	.086	1.412	.953	2.092
T pathologic, T2-4		1.000		
T1	.105	.657	.396	1.091
Tis	.031	.165	.032	.844
N pathologic N0		1.000		
N1-3	.115	1.580	.894	2.791
Grading G1		1.000		
G2	.238	.720	.417	1.243
G3	.301	.630	.262	1.513
Lymphvessel invasion, L0		1.000		
L1	.337	1.356	.728	2.526
Vascular invasion V0		1.000		
V1	.329	1.780	.560	5.662
Hormonal receptor status, positive		1.000		
Hormonal receptor status, negative	.036	2.241	1.056	4.756
Her2/neu, positive		1.000		
Her2/neu, negative	.049	.500	.251	.996
Ki67, low risk < = 15%		1.000		
Ki67, high risk > 15%	.995	1.002	.574	1.748
associated ductal carcinoma in situ, yes		1.000		
associated ductal carcinoma in situ, no	.003	.414	.231	.741
Neoadjuvant chemotherapy, yes		1.000		
Neoadjuvant chemotherapy, no	.190	1.975	.714	5.459
Preoperative needle marking, yes		1.000		
Preoperative needle marking, no	.744	.917	.545	1.542

*OR odds ratio

**Table 4:** Odds ratios (OR) for risk of R1 after Margin Shaving versus standard surgical procedure in patients with BCS with invasive breast cancer and carcinoma in situ. Binary Logistic regression analyses in total cohort and subgroups

Category	Group	Univariable logistic regression				Multivariable ^*^ logistic regression			
		*p*	OR*	Lower 95%-CI	Upper 95%-CI	*p*	OR*	Lower 95% CI	Upper 95% CI
Total		.887	.962	.563	1.643	.738	1.104	.620	1.965
Histological type	Inv. ductal carcinoma	.722	.890	.467	1.694	.723	.881	.438	1.772
	Inv. lobular carcinoma	.857	.857	.160	4.583	.391	3.041	.239	38.723
	Carcinoma in situ	.737	1.306	.274	6.222	.573	1.924	.198	18.751
Invasive ductal carcinoma only									
Age at diagnosis (years)	< 50	.188	2.700	.616	11.835	.122	6.246	.611	63.842
	50–59	.743	1.214	.381	3.874	.410	.538	.123	2.353
	60–69	.054	.227	.050	1.027	.072	.168	.024	1.171
	≥ 70	.521	1.592	.385	6.586	.351	2.251	.408	12.414
T pathologic (pT)	T1	.065	.247	.056	1.090	.057	.214	.044	1.050
	T2-4	.100	2.244	.857	5.874	.172	2.580	.662	10.053
N pathologic (pN)	N0	.861	1.079	.459	2.538	.832	.902	.348	2.338
	N1-3	.270	.407	.083	2.009	.120	.114	.007	1.758
Grading	G1	.122	.193	.024	1.552	.120	.149	.014	1.637
	G2	.936	.965	.407	2.292	.826	.898	.344	2.347
	G3	.477	1.757	.371	8.317	.663	1.890	.108	32.994
Her2/neu	positive	.657	.596	.061	5.858	.354	.303	.024	3.793
	negative	.707	.876	.439	1.750	.531	.782	.361	1.691
Associated ductal carcinoma in situ	Yes	.916	.932	.252	3.444	.944	.951	.238	3.797
	No	.688	.858	.407	1.811	.650	.827	.365	1.876

*Adjusted for age at diagnosis, histologic type, breast side, T pathologic (pT), N pathologic (pN), grading, lymphvessel invasion, vascular invasion, hormonal receptor status, Her2/neu status, Ki67, associated ductal carcinoma in situ, neoadjuvant chemotherapy and preoperative needle marking

## Discussion

The present study compared 80 patients with CS during BCS and 511 patients with standard surgical strategy regarding residual tumor rates in a multivariable regression model. No significant difference between both surgical techniques regarding risk of residual tumor was found (*p* = 0.738). A tendency for benefit from CS was only seen in patients with invasive breast cancer who were between 60 and 70 years old (*p* = 0.054) and patients with smaller, T1-tumors (*p* = 0.065). Though, statistical significance was not reached. Our study suggests that there is no benefit from CS in comparison with the standard technique regarding re‐excision rates.

In 26,9% of the cases in our study histopathological positive tumor margins were found, which is among the international range of re-excision rates of 20–40% [18].

However, previous studies described a statistically significant reduction of re-excision by additional CS and a lower rate of positive margins of lumpectomy specimen than BCS alone. Chapgar et al. described a reduction of margin positivity in BCS with CS compared to standard BCS in nearly 50% of the patients (*p* = 0.01) from a total cohort of 235 patients in his randomized controlled trial as well as decreased re‐excision rates by CS (*p* = 0.02) [10]. Our conclusion that CS is not significantly beneficial in comparison to standard procedure was similar to the one of Chen et al., who claimed in his randomized controlled trial (*n* = 181) that neither re‐excision rates (*p* = 0.65) nor R1-status (*p* = 0.07) were significantly reduced by CS [22]. This may vary from Chapgar et al. randomized controlled trial due to different characteristics of patient cohorts (i.e. smaller breast volumes in Asian population) [9]. According to Wang et al., a reduction of R1-status and re-excision rates of 59% was reached by CS [7]. Wang et al. performed a systematic review and meta-analysis which included 24 retrospective, non‐randomized studies and two randomized controlled trials. This meta-analysis shows the wide heterogeneity among different studies. Reduction of re-excision rates and R1-status was also not as high in other studies, i.e. Hequet et al., who claimed that re‐excision was avoided even by 25.3% in a retrospective study of 99 patients [23]. Chen et al. found no significant reduction by CS neither in re‐excision rates (*p* = 0.65) nor in margin positivity (*p* = 0.07) [22, 24]. No effect of CS on re-excision rates was shown neither in the retrospective study patients of So et al. [25] neither in the retrospective, case-matched study of Pata et al. [24]***.*** Feron et al. claimed that re‐excision was avoided in 24% of the patients due to CS. CS reduced false positive margin status and contributed to a more accurate margin examination, i.e. for multifocality [26]. Our study was restricted to invasive ductal carcinoma. Invasive lobular carcinoma or ductal carcinoma in situ were not analyzed due to the smaller size of cohorts. Though, subgroup analysis was decisive in other studies, which described positive effects of CS on margin positivity only in certain subgroups, such as invasive ductal carcinoma [24, 27], multifocal tumors [26], luminal A, B or triple negative tumors [28] and lobular carcinoma [29]. Others, such as Heiss et al. found no reduction of re‐excision rates in ILC or DCIS, which have been claimed to have higher risk for re‐ excision (30). In our study the risk of R1 status was threefold higher for DCIS in comparison with invasive ductal carcinoma, equally in patients with BCS with CS or standard procedure with an OR of 3.413 (95%, CI 0.72–16.11, *p* = 0.121). A reason could be the irregular pattern of growth of DCIS. As described previously, our findings from multivariable regression analyses showed that CS seemed to have a benefit with almost statistical significance (*p* = 0.072) in patients from 60 to 69 years old as well as smaller T1 tumors, which showed a tendency of reaching R0-status more often with CS (*p* = 0.057). For other analyzed variables such as nodal status, grading, HER2neu status or associated DCIS no significant difference between both surgical methods regarding the risk of final R1-status was seen. The lack of a standarized, tangible definition of exact volume and width taken in different surgical strategies may be the cause of wide heterogeneity in different studies. In all studies, the resected tissue volume was performed by the surgeon's decision. The present study has several limitations. The different contribution of CS among all five surgeons was a limitation of our study. Among patients receiving CS surgeon 1 dominated with 61.3%, whereas the others performed this technique in 13.8% of the cases at most. However, surgeons belonged to the same team, were trained in the same way and were experienced. As this study was retrospective, recall bias and selection bias were unavoidable.

In conclusion, women with invasive ductal carcinoma aged between 60 and 69 years and patients with small tumors seemed to have a slight benefit from cavity shaving regarding risk of residual tumor. Apart from that, there was no statistical difference between cavity shaving and standard procedure. CS is a surgical technique which has a controversial benefit in reduction of re‐excision rates, the general use of CS in BCS cannot be recommended. Further prospective randomized controlled trials are needed.

## Data Availability

Not applicable.
